# Moving Beyond the Gay Metropolises: Lessons Learned from Stellenbosch

**DOI:** 10.1007/s12132-023-09490-2

**Published:** 2023-03-16

**Authors:** Gustav Visser

**Affiliations:** Stellenbosch University, Stellenbosch, South Africa

**Keywords:** Gay men, Homonormalisation, Stellenbosch, South Africa

## Abstract

After roughly 20 years since the emergence of urban scholarship in same-sex sexualities in South Africa, it is worthwhile considering how some of the concerns that originally animated that scholarship have evolved and also how such concerns are today reflected differently away from primary cities (where much earlier research was conducted). In this commentary, I explore the unique history of Stellenbosch, a university town/secondary city 50 km away from Cape Town. Stellenbosch’s own unique history of—and recent developments with regard to—public (male) same-sex expression help set into relief earlier scholarship and also points towards some future research questions that may also be applicable elsewhere on the African continent. While, as made clear, Stellenbosch is in some key instances unique in terms of its sexualized and raced history both in South Africa and the wider continent, its position as what we might increasingly want to frame as a secondary city, its particular racial composition, and also its changing spaces of socio-sexual interaction since the COVID-19 pandemic gesture towards key areas of potentially generative wider research interest.

## Introduction

“Daar is te veel gays hier” (There are too many gay people here)

I recently overheard this remark uttered at a bar in Stellenbosch. The venue was not particularly trendy, perhaps somewhat eclectic in style, and attracted a rather broad range of patrons, both in terms of age and sexual orientation. Ultimately, however, the venue was a predominantly white, heterosexual space. I was at the bar that evening to meet up with my gay friends since it was a Wednesday, which was when we would usually congregate there. The comment expressed by one young, white, Afrikaans man to another made me question how a space that was by no means a gay space and that was populated by an overwhelmingly white and mainly straight crowd seemingly “offended” these two men. (There is of course context to be applied to the scenario—this experience transpired during one of the COVID “curfew phases of 2021” in South Africa, wherein there were, in effect, not that many leisure places to go to for anyone, including these young men. In fact, many similar leisure spaces during this time had shut down their operations, and many never opened again.)

Yet the point I wish to make is that the “spontaneous reaction” of these men was one that seemed to suggest that they found themselves in a space that, by not being seen as wholly heterosexual, made them feel uncomfortable enough to move on. Was it therefore possible that a space I would view very much as a heteronormative space was in fact experienced as “too gay” (or not straight enough)? And what would this remark in turn mean for existing work, and potentially future work on, African urban sexualities?

These questions made me reflect on earlier research I conducted and related discourses around the preservation of visually consolidated, protected, and defended spaces for the more broadly framed queer community.^1^ Much of the debates concerning these issues are derived from the experiences of largely white gay men and their relationship to consolidated physical and symbolic space(s) in Western contexts. As Harry Britt, a gay political leader in San Francisco, once commented many years ago to the sociologist Manuel Castells, “when gays are spatially scattered, they are not gay, because they are invisible.” (Castells, 1997, 272). I am not sure how many of the gay people many decades later and thousands of miles away in Stellenbosch on that Wednesday night would have agreed with Britt’s claim. We may wish to frame a potential disagreement first in relation to the fact that the gay people who were in that bar on that Wednesday in Stellenbosch were not entirely spatially consolidated; the presence of significant numbers of heterosexuals points to that. Yet at the same time, the gay people in the bar that night were not entirely spatially scattered and therefore invisible; despite large numbers of heterosexuals in the establishment that night, there was still a feeling for some heterosexual patrons that the bar was indeed “too gay”. In this commentary, I wish therefore to consider both Britt’s comment and the comment by the two men in a bar one Wednesday in light of the experiences of gay men, and gay white men in Stellenbosch more specifically, and their place in a partially homonormalised urban setting. There are very considerable limitations in this aim, for it pivots solely around gay white men and could seem out of step with a continent that is neither majority white nor gay. However, as discussed in the conclusion to this intervention, this narrative might also provoke investigatory imaginations to rethink lived gay realities in other urban African places and spaces.

## Situating this Reflection

Many years ago, I started on a journey of thinking through the geographies of gay male expressions of identity in urban space in South Africa (Visser, 2002, 2003a, 2003b, 2008a, 2008b, 2010, 2013). This was driven by the notion of how gay men in particular created dedicated places and more broadly spaces that could act as a foil against often hostile and sometimes violent heterosexual environments. In Western contexts, these debates in social sciences stretch back to the 1970s, although their practice they have been evident for much longer (Doan, 2015; Hughes, 2006; Waitt & Markwell, 2006).

My earlier investigations at the beginning of the 2000s tracked the emerging empirical realities of consolidated “gay space” and the notion of Cape Town as “Africa’s Gay capital”. Subsequently, detailed investigations have expanded upon and significantly refined my initial observations (e.g. Matebeni, 2016; Rink, 2016; Tucker, 2009). My interest in this investigatory nexus followed on from my academic journey, which began in London with its very well-established gay scene, which was then refocused to Johannesburg with the seeming emergence of a gay leisure node (which never fully developed in the same way as places such as Soho in London but rather took on their own trajectory—see, for example, Khuzwayo 2023 this issue), then to Cape Town, then to Bloemfontein during my time there, and finally to Stellenbosch. Through this roughly 2-decade period, I have charted and witnessed great changes in forms of visible sexuality-based expressions in urban environments at numerous levels in various places. Some of this was owing to changing legislation, changing ways in which gay lives are included or not in some parts of society, and how those identities are lived and (re)negotiated.

In contrast to my earlier work on Cape Town and the very well-researched De Waterkant gay village in particular, this investigation is concerned with Stellenbosch as a space that does not share the gay development trajectory followed by other places that I had previously studied but which rather likely has far more in common with a number of other urban places around the world and potentially across the African continent. In this intervention, I set out to explore some themes of “gay visibility”, which, in the end, circle back to the remarks of the two young men in the bar. To be clear, in large part due to the short nature of this intervention, I am not setting myself the task of exploring how spaces and identities are “queered”. Rather than push in the direction of post-structuralist and deconstructionist critique, I am instead interested in how the power relationships of gay versus “straight” have been renegotiated such that gay normative acceptance now gestures towards something perhaps more visible but still heavily dependent on race and class positions. The point I wish to make is that this new formation does not occur in fixed and dedicated “gay space,” as some of the earliest work on the relationship between sexuality and urban space in the global North suggested. By way of conclusion, I revisit a framework I explored several years ago, namely that of homonormativity and homonormalisation, to consider not only how such terminology remains sometimes tethered to research on regressive outcomes for individuals with same-sex desire but also the productive potentials for such a framework to understanding the complexity of the relationships between sexuality and space in future research.

## Stellenbosch—an Invisible Gay History

A broader historical context should first be provided to position my remarks. Most South Africans, indeed also individuals elsewhere, conjure Stellenbosch as a town (while today it is actually a secondary city^2^), a historic space imbued with myths about its symbolic meaning to white Afrikaner nationalism.^3^ As is the case with many such narratives, the details are far more complex. I think it fair to say that it was both a closed and open space all at once, and sexuality would have been part thereof. Even if only the clichés of famous gay artists associated with the university and the town is any measure, there were ample examples of gay lives lived.^4^ These were gay lives, however, mostly concealed for decades. The point is, and I would come to learn, they were nevertheless there, as they were also in fact elsewhere in urban Africa (see Luongo, 2007; Tamale, 2011). This leads me to the next point.

First and foremost, there is a need to acknowledge the fact that gay visibility in Stellenbosch is devoid of dedicated geographical or urban studies. I would argue that there are two main reasons for this scholarly oversight. Firstly, places of consolidated gay space never developed in Stellenbosch, and secondly, there has never been geographical or allied scholarship at Stellenbosch University that would have remotely focused on the geography of gay/queer sexuality (Visser & De Waal, 2020). I would suggest that perhaps the former is more important as a starting point to propose an active research programme in this regard going forward. Before doing so, however, I furnish personal notes on gay places/spaces from the early 1990s.

In common with most gay geographies worldwide, gay visibility was carefully negotiated in both public and private spaces in Stellenbosch. Personal networks were only for those in the “know”, mostly by chance encounters or via “referral”. This was a trend long before I arrived at Stellenbosch in the early 1990s. I knew gay men personally via my engagement with older gay men as a waiter at a local restaurant—they tipped me well and sometimes not that discretely. These older men did open a door to a very private and hidden gay world. It was also a world of caution—these men were of the few that had somehow managed to evade HIV, although many of their friends had not or had already passed at that time. Their friendships were ones that signalled caution, and a lesson taken seriously by many of the publicly invisible young gay Afrikaans men in Stellenbosch that they knew. These older men guided us on where to go and how to act but also informed us of the rules of engagement with different types of leisure spaces. These rules of engagement could also be seen as ground rules, which were very much of edited gay performativity in white heterosexual environments. Any dissonant ideas played themselves out in the far larger neighbouring city of Cape Town, where there was an emerging gay village in the form of De Waterkant. Your “real gay self” was to be explored there, not in Stellenbosch.

My personal exploration of the link between gay identity and space during the early 1990s in Stellenbosch was at places such as *De Akker* and *Rustic Café*. During the period, one might describe these spaces as “arty” with “alternative music” and as “liberal”—certainly liberal in terms of the patronage of political reform-oriented students and student leaders in a late Apartheid dispensation. Along with this came a strong grouping of young, working adults. I think with this type of territory came some greater acceptance of those men and women who did not quite fall into the overtly aggressive displays of their heterosexuality. In turn, this acted as a kind of foil^5^ against the sporting jocks supporting these places/spaces. Notwithstanding, gay men, whether young or old, needed to fit in with intimacy, not for public display, even in these spaces (so very much a matter of editing of gay performativity). What is noteworthy is the fact that there were also ground rules set in these spaces prohibiting all (at least overt) violence against (white) gay patrons. On the occasions that an incident did occur, i.e. when there was some gay bullying and fighting, it was the straight guys that were thrown out of the venue.^6^ In the end, those particular heterosexuals simply did not support these venues.

As the 1990s progressed, there emerged the rise of the De Waterkant as a prominent gay space in Cape Town. Owing to the small size of the population of Stellenbosch at the time, there were no real dance clubs, so clubbing in Cape Town was usually a much anticipate outing (physically and symbolically). In some ways, this only served to undermine the creation of agglomerated “gay spaces” in Stellenbosch. Some of us, including myself, tried to establish a more structured gay leisure life in Stellenbosch by organising parties at local bars and drawing on the small circle of gay men we knew—most of whom were not officially out. This led to a rather closed, albeit expanding group, of mainly closeted gay men but did not do much to really provide gay visibility, let alone something approaching queer visibility. These attempts at gay space creation/visibility were a spectacular failure since, in reality, there was little interest to be visibly gay in the then still relatively small university town, wherein there posed the risk of being outed even by association, and with far greater anonymity with Cape Town down the road, seemingly more attractive. An analytic question would be how gay life was lived in other racially segregated parts of Stellenbosch then and the places and spaces generated, or not. This extends to the continent too.

What also has to be acknowledged is that the majority of my time in Stellenbosch was what was to come to be known as the late apartheid era. My life world as a result was very much a white one, segregated from other racial and classed communities in countless ways. In terms of friendships or acquittances, I lived in a monochrome world. Considerations of “other” gay lives were not on the radar—I was trying to find my own footing in a heterosexual world. I did not for a moment consider black or coloured (an initially very problematic apartheid construct developed by colonial administrators and later “refined” by the Apartheid government (see Sparks (1990) subsequently re-appropriated and now deployed as one of the population categories in the post-apartheid South African national census) lives. Race was just an “obvious category”. Yet, those lives were lived in South Africa, and something the academic record needs to more fully account for (see, for example, Tucker, 2009). Yet here again, what scholarship that has emerged has tended to focus on the far larger Cape Town and its De Waterkant gay village, a space it seems that had gravitational pull both for young gay men in Stellenbosch and for academics wanting to explore the intersection of race, class and sexuality.

On the one hand, then, the study of Stellenbosch in relation to the intersection of race, class, and sexuality is a study clearly needed and yet to occur. My own trajectory outlined here presents but one particular narrative of a clearly far more complex tapestry of wider South African urban sexuality experiences. And just as scholarship on Cape Town has highlighted the richness of that tapestry, so too could scholarship considers such richness in relation to places such as Stellenbosch, which remain both under-researched and which could prove enlightening in relation to their physical proximity (and gravitational pull) of larger primary cities such as Cape Town. The discussion above, hopefully, can be seen in the light of the pressing need for more research on urban sexualities on the continent that do not take as their starting point only large primary cities. To only focus on large primary cities would be to potentially repeat the challenges faced by urban sexualities scholarship in the global North, where attention was initially overtly on places such as New York, San Francisco, and London. Yet on the other hand, while a lack of historical and contemporary work on diverse racially-defined groups can indeed rightly be seen as a key oversight, both in my own thinking in the early 1990s and also in early scholarship on sexuality in South Africa, there may still be applicability about thinking about the history of “whiteness” in the context of urban sexualities and putting it into conversation with the experiences of other groups elsewhere on the continent today. This is a point I return briefly to in the conclusion. But before then, I turn to contemporary gay Stellenbosch and its evolving relationship to visible and invisible forms of same-sex expression.

## Gay Stellenbosch Now

The narrative so far has looked at a particular moment in Stellenbosch’s history, as ostensibly straight leisure spaces increasingly during the first years of the post-apartheid dispensation included individuals who, to limited degrees, were at least partially open about their sexualities. And the place has continued to evolve. It is, after all, now the headquarters to a range of South African and international holding companies.^7^ With them has come well-educated and wealthy executives and managers, often in the knowledge and financial economy. Equally, the student body has also continued to evolve. No longer is Stellenbosch a majority Afrikaans-speaking university. The primary feeder schools for the university are very different now from what they were a few decades ago.^8^ Consequently perhaps, curated behaviour, when confronted with sexuality differences, has also continued to shift. Stellenbosch has also developed a major leisure scene owing to a tourism drive attracting vastly more international students. The “town” has also for some time now been repopulated by “semigrators” from provinces elsewhere in the country, such as Gauteng and KwaZulu-Natal, who seek different things.^9^ With this, a more open community in terms of sexuality has developed.

Over the past 2 decades, there were at first subtle changes that took place to which I would like to draw attention. The changing demographics of Stellenbosch have gone hand in hand with an evolving high-end consumption market increasingly at odds with overt hostility to same-sex sexualities. In its simplest terms, competition and the growth of venues for patrons, combined with the rise of new types of leisure spaces, have shifted the needle of potential sexuality-based hostility. Be “nice” or lose the patron. The rise of the hipster movement, for example, brought with it an appeal to gay men, for whom their visible inclusion was seen to add to venues’ appeal. The same can be said for the rise of wine bars over the past 15 years. Whilst the introduction of these new establishments does not take away from the fact that these are white and wealthy spaces, they do provide a type of context that requires something other than overt hostility to those that are not heterosexual. Again, there is an opening for those that move and/or are outside some sort of the heterosexual norm. As such, there is a simultaneous movement of the gay narrative.

Today, there are groupings of young gay men in Stellenbosch that I imagine have never set foot in a gay bar or club. Some of them find it curious that they would want to socially separate themselves from their straight friends. Their sense of seeking community is different. The idea of seeking sex in bars or clubs is seen as outdated and inefficient. Cruising, that activity that sustained so many gay bars in the past, has gone online, a shift made all the more apparent by the COVID lockdowns. This statement cuts in different directions. These kinds of worldviews are in many ways different from those seeking their gay self in space and place and the types of debates that predominated the foundational texts of the initial gay space/place debates of the past and primarily in the global North (Gorman-Murray, 2007; Waitt & Markwell, 2006; Vorobjovas-Pinta, 2021). Yet it also potentially robs them of intergeneration gay learning and living—the impact of which will still need to be determined.

## Conclusion

The two young men that entered a bar and found themselves outside of “their” heterosexual place/space of comfort in a place that was certainly not a gay bar offered the starting point for this intervention. While, as outlined, the existence of solely “gay spaces” has been an anathema to Stellenbosch, so too, it would seem, has the existence of solely “straight” places also increasingly become so too. No doubt, the rupture caused by the COVID-19 pandemic and its strict and long lockdowns in South Africa accelerated this process. The lockdown and its after effects have forced more people into fewer places, aiding, in my view, the homonormalisation of a number of ostensibly heterosexual leisure spaces.

So what does this mean for future research on African urban sexualities? To be clear, the above short history could well be framed as that about white gay men in Stellenbosch and is therefore not representative of much. The contours of race, sexuality and space, after all, remain fundamental to scholarship on sexuality, and the urban in South Africa and further afield. However, I would like to suggest that in some key ways, the experience and history of Stellenbosch, as outlined here, may well be worth keeping in mind as sexualities scholars start to look at other, lesser well-known urban environments across the continent. There are several ways to consider this.

First, and most clearly, scholarship on urban sexualities, be it in Africa or the global North, has tended to overtly focus on the “big” cities and those spaces that have been most “visible”. But the story of Stellenbosch shows us that the impact of those spaces (for example, Cape Town’s gay village, enabled and furthered by South Africa’s liberal constitution) has also affected neighbouring cities and towns. As Livermon (2023) in this special issue also outline, the “reach” of cities such as Cape Town and Johannesburg may well extend far beyond their own municipal boundaries in extremely diverse ways.) Furthermore, the existence of delineated “gay spaces” such as De Waterkant in Cape Town, we may wish to consider, is not only not emblematic of the interface between sexuality and space more broadly but may also help drive and legitimate other forms of socio-spatial interaction that appear far less “visible” in South Africa and elsewhere. How much socio-spatial interactions manifest for diverse groups and the relationships such forms of interaction have in relation to other potentially larger cities is a topic that still needs further exploration.

Second, the development of spaces that appear to have broken down a neatly defined binary between “gay” and “straight” also reminds me of my earlier work on homonormativity. As some scholars from the global North have long argued, the supposed gains made in terms of sexuality-based equalities may well mean the eventual “end of gay” created by a collapse of the heterosexual/homosexual binary (Sullivan, 2005; see also Podmore, 2013). (In South Africa, one could well argue along a similar trajectory, with the country having the most liberal constitution on the continent with respect to same-sex rights.) Yet additionally, other scholars have pointed out that such assimilation has tended to overtly focus on those with the class, race, and gendered privilege to in effect disappear within a sea of normative (hetero)sexuality (Duggan, 2002; Nast 2002). For these scholars, a new form of normativity, that of homonormativity, became that which delineated those who could assimilate. Left beyond the bounds of homonormativity are marginalised and/or “radical” queer subjects, who cannot—or do not want to—assimilate. Drawing on my earlier work (Visser, 2008b), I would however caution here at such a use of the concept of homonormativity. Between radical queer subjects and subjects that could be read as capitulating towards a form of heterosexual assimilation, I have charted in this commentary another way of reading how gay male subjects interface with ever-changing urban space. The lack of delineated gay spaces, as ruptures in heteronormative environments, should not necessarily be seen as simply assimilative. Drawing on Podmore’s (2013) work on homonormativity, we may wish to see such framing as “too neat”. Instead, we should be cognisant of the struggles, erasures, moments of qualified acceptance, and—as exemplified by the comments such as “Daar is te veel gays hier”, moments of continued rupture with heteronormativity—as both historical and ever-present, even among those with the gendered, classed, and raced privilege of many patrons in bars in a university town such as Stellenbosch. While in no way denying or attempting to underplay the multiple privileges of those who have the time and resources to socialise in leisure spaces in Stellenbosch, we must remain cautious of erroneously erasing local histories and local complexities in the search for simplistic theoretical congruities. This, one could possibly argue, speaks also to the wider remit of this special issue to think through how greater empirical research is needed to drive theorisations from the South, rather than apply theorisations from the urban North unproblematically elsewhere.

Third, my ambitions going forward are questions surrounding the other histories and realities of lived gay lives in South Africa and elsewhere in Africa in smaller urban locations—not just the large cities. I would anticipate much evidence will be found, if we know how to look for it, of gay lives that are carefully constructed, perhaps invisible to the naked eye (see also Ombagi (2023) and Gevisser (2023) in this issue). What I would caution against is the idea of seeking out a familiar Western interpretation of gay life in relation to consolidated physical space. Gay sexuality in place and space is always locally negotiated (Tamale, 2011). At the same time, however, future research may also want to consider how, in particular acute ways, the experiences of Stellenbosch in relation to raced and classed privileged may indeed play out also in unique constellations elsewhere across the continent. For example, left to explore are the negotiations that may be ever present in various “expatriate” communities across the continent, often made up of policy, aid, or development workers from the global North, and the way they may speak to or elide local forms of sexuality-based liveability and sociability.

What I would ask us to resist, however, is to talk about “gay in Africa” and any relationship to space as if there is some sort of universally binding meta narrative to be found. We need to remember that gay life does not “look the same” everywhere nor for everybody, and I think the idea that there will be an expansion of gay space per se seen in many European or American cities seems unlikely and indeed potentially anachronistic. The widespread introduction of technology plays a key role in this regard. The kinds of questions I have posed require empirical engagement elsewhere in South Africa and indeed across the continent, as I do wonder whether recently, in a Black or coloured (etc.) community, two young men going to a bar have said, “Daar is te veel gays hier” (There are too many gays here) and have moved on because some places and spaces have indeed, perhaps in part, become homonormalised.

## Footnotes

1 I need to be clear that the reflections here are concerned with white gay as opposed to far broader queer sexualities. The main difference between queer and gay is that queer is a wide term for sexual minorities that are not heterosexual or cisgender. It can refer to entire LGBT communities or to particular groups whose existence may “trouble” or destabilise taken-for-granted identity categories (Wilchins 2004). Gay, meanwhile draws inspiration from earlier communities and scholarship on homosexuality as defined in a binary and often times oppositional relationship with heterosexuality. Analytically these descriptors may share some overlap, but should be carefully negotiated and not uncritically conflated (Anderson and Knee 2021).

2 The underlying analytic point here is that urban theory building and issues such as gay and queer identity relationship to urban space cannot only be framed by primate or large urban complexes. Much of urban life is not necessarily lived in metropolitan areas, and scholarship suggests that urban growth across Africa in the coming decades will not occur solely in primary cities but rather also across a host of secondary cities.

3 This symbolic meaning ascribed to Stellenbosch was/is reinforced by systems of governance (prestigious education at all levels, culture and religion, entertainment, a very strong services and technology economy as well as, property markets) that still sustains majority white and Afrikaner interests (see Du Toit 2019) and reflected in the physical and symbolic development of its urban spaces.

4 For example, Nathaniël (performing arts, authors, and television personality), Joan Hambridge (celebrated author and outspoken literary critic), and Casper de Vries (comedian).

5 I use “foiled” here in the sense that the majority of heterosexuals in these spaces were, at least in part, accepting of gays and lesbians and willing to “stand up” for them against homophobic men/women.

6 I would argue that in many other places, it would have been the gay men asked to leave.

7 Mediclinic (international hospital group); Remgro (international investment holding company), Distell (one of the largest wine and spirits holding companies in the world), Capitec (South Africa’s largest banking group by clients) and PSG (financial services).

8 Proportionally, Stellenbosch University now attracts the largest number of learners from South Africa’s elite private and public schools that are often English-speaking and wealthy.

9 See Du Toit (2019) on the changing patterns of elite migration to the Cape Winelands generally and Stellenbosch in particular.
